# Development of a New FT-Raman Method for the Investigation of Cinnamon Authenticity

**DOI:** 10.3390/foods15081311

**Published:** 2026-04-10

**Authors:** Konstantinos Chatzipanagis, Ana Boix Sanfeliu

**Affiliations:** Directorate-F Health and Food, Directorate-General Joint Research Centre, European Commission, Retieseweg 111, 2440 Geel, Belgium; ana.boix-sanfeliu@ec.europa.eu

**Keywords:** Raman spectroscopy, multivariate statistics, screening, cinnamon authenticity

## Abstract

The price of *Cinnamomum verum* (*Ceylon cinnamon*) is higher than that of other cinnamon varieties known as cassia cinnamon and/or *Cinnamon camphora*, which can trigger fraudsters to perform partial or total substitution of the former by the latter types, especially in ground cinnamon products. In addition, substitution of cinnamon bark by different parts of the plant (e.g., root, leaves) and/or introduction of inorganic matter in any cinnamon variety can also occur, increasing the overall risk of fraud related to spices, which are among the most vulnerable food commodities. This work reports the development of a novel Fourier transform (FT) Raman spectroscopic method combined with principal component analysis (PCA) applied to ground cinnamon, as an analytical tool to detect suspicious samples related to the aforementioned fraudulent practices. The findings of this new analytical approach were supported by published results from experiments on these samples using confirmatory techniques, such as X-Ray Fluorescence (XRF) and Gas Chromatography–Mass Spectrometry (GC-MS), cited in this paper.

## 1. Introduction

Cinnamon is an old spice that belongs to the genus *Cinnamomum*, which is part of the Lauraceae family and includes more than 200 species in Southeast Asia, China, and Australia. Commercially available cinnamon comprises the dried inner bark of the tree *Cinnamomum verum*, indigenous to Sri Lanka, and is considered the highest quality cinnamon, with a mild and sweet flavour, known as *Ceylon cinnamon*. On the other hand, lower quality cassia cinnamon has a stronger flavour due to higher cinnamaldehyde content compared to Ceylon, with different species originating from China (*C. cassia*), Vietnam (*C. loureiroi*), and Indonesia (*C. burmanii*) [1]. Apart from Sri Lanka, which is the major producer and exporter of bark and leaf oil, other regions such as the Seychelles, Madagascar, and India also produce true cinnamon bark of high quality, albeit in smaller quantities.

Cinnamon has been utilized in cooking and traditional medicine for hundreds of years [2,3], while the current total production by China, Vietnam, Indonesia, Sri Lanka, and Madagascar together reached 238,105 tons in 2023, representing more than 99% of the world’s production [4]. In 2023, the European Union imported 13,300 tons of cinnamon, placing it fifth among the most imported spices, with the majority coming from Vietnam [5].

In terms of legislation, cinnamon available in the EU market must comply with several regulations, among which is Regulation (EC) No 1334/2008 that sets a limit on the levels of coumarin, ranging from 5 mg/kg in desserts to 50 mg/kg in bakery products containing cinnamon [6]. Nevertheless, there are no legal requirements applied to the amount of coumarin naturally present in cinnamon, despite the scientific opinion given by the European Food Safety Authority (EFSA) on Tolerable Daily Intake (TDI) levels and recommendations from the food authorities of various Member States (e.g., France, Germany) [7].

Coumarin is a chemical substance that exhibits hepatotoxic effects, with a TDI of 0.1 mg/kg bodyweight [7,8], and is mainly found in cassia cinnamon, while its presence in *Ceylon cinnamon* is minor. Since cinnamon is a very prominent ingredient for a wide range of products (e.g., milk-based) used in food, it is anticipated that demand will rise over the following years, with a global compound annual growth rate (CAGR) of up to 12.4%, with the European market aligning with this trend [9,10]. This growing demand has already caused an increase in the price of cinnamon across Europe at an annual rate of 10% since 2017 [11]. In addition, taking into account that the Ceylon species is approximately two times more expensive than cassia and other species, the rising consumption of cinnamon may instigate fraudulent practices such as partial or total substitution of Ceylon with cassia in order to increase profitability, which can subsequently impose health risks linked to excessive intakes of cinnamon that may contain substances such as coumarin. Another type of fraudulent practice could also involve the replacement of both Ceylon and cassia cinnamon with other cheap cinnamon species, such as *Cinnamon camphora*, as well as the substitution of cinnamon bark with other parts of the plant (e.g., root, leaves, flowers, seeds). It is noted that although Ceylon and cassia species can be easily differentiated by visual inspection when purchased as bark due to their different morphologies, this differentiation becomes cumbersome for ground cinnamon, making ground products more susceptible to fraud.

In light of the above, it becomes clear that the need to develop and use analytical tools for the detection of cinnamon adulteration in ground cinnamon is of paramount importance to ensure consumer protection. In this regard, vibrational spectroscopy has been applied to study cinnamon speciation as well as the potential substitution of *Ceylon cinnamon* with cassia. Fourier transform infrared (FTIR) spectroscopy combined with multivariate statistics has been previously used on thin films coated with cinnamon essential oil [12] or directly on cinnamon powder samples [13,14,15] to study the differences among cassia and Ceylon species, as well as the substitution of the latter with the former. In addition, FTIR was also employed for the first time to determine the adulteration of *Ceylon cinnamon* with cinnamon spent (waste) via the preparation of several admixtures of the individual cinnamon species at varying ratios [13]. Other studies reported the use of near-infrared (NIR) spectroscopy combined with multivariate statistics to detect the adulteration of *Ceylon cinnamon* with cassia [15,16,17] as well as with extraneous substances such as black pepper and/or clove [18]. Nelson et al. applied surface-enhanced Raman spectroscopy (SERS) on the essential oil extracted from Ceylon and cassia cinnamon samples to explore the spectral differences among the volatile compounds present in the two species, such as coumarin in cassia (absent or very low in Ceylon) and eugenol in Ceylon (very low in cassia), that can be employed as chemical markers to detect cinnamon adulteration [19].

However, most of the aforementioned studies were conducted either on cinnamon mixtures prepared from a limited number of cinnamon samples and/or on cinnamon samples purchased from a few marketplaces, which may limit the representativeness. Moreover, these studies mainly focused on the substitution of Ceylon with cassia and largely relied on infrared spectroscopy (mid-IR and Near-IR). No prior work assessing the potential substitution of cassia and *Ceylon cinnamon* with *Cinnamon camphora* and/or other parts of the plant has ever been reported using vibrational spectroscopy.

Hence, this study introduces a novel untargeted FT-Raman approach integrated with PCA, presenting some key innovations: 1. the method is applied directly on ground cinnamon and a large set of commercial samples (>100) from multiple European countries is analyzed, providing broad market representativeness; 2. beyond the substitution of Ceylon by cassia, the proposed approach demonstrates the capability to identify suspicious cassia and *Ceylon cinnamon* samples potentially substituted with *Cinnamon camphora*, other parts of the plant, or even inorganic matter.

Notably, the detection of these types of substitutions represents an important novelty and is particularly well suited to Raman spectroscopy, as shown in the following sections and corroborated by complementary analytical techniques including XRF and GC-MS [20,21].

## 2. Materials and Methods

### 2.1. Cinnamon Samples and Essential Oil Compounds

One hundred and four commercial cinnamon samples were bought in several European countries, such as Austria, Belgium, Bulgaria, France, Greece, Italy, Malta, Germany, Slovakia, Spain, Serbia, and the UK (see Table S1). The samples were purchased at various supermarkets and small retailers to prevent the use of samples belonging to the same batch/original producer. Six samples were sold as bulk, and the rest were pre-packaged and branded as registered trademarks. Fourteen samples were purchased online, all of them commercialized by registered trademarks.

Based on the information provided on the labels, 44 samples were labelled as *Ceylon cinnamon*, 4 as *C. burmanii*, 1 as *C. burmanii*/*cassia*/*loureiroi*, 1 as *C. aromaticum*, and 11 as *Cassia cinnamon*; no further information on the botanical variety of the remaining 43 was available. In terms of the origin of the cinnamon, 22 were from Sri Lanka, 6 from Madagascar, 7 from Vietnam, 2 from India, 3 from Indonesia, 2 were a mixture of cinnamon from Sri Lanka and Indonesia, and 1 indicated that it originated from the Tropics. The rest of the specimens were either indicated as “outside EU” or “non-EU”, or no information about their origin was given. Seventy samples were ground (according to the labels, 27 were *C. verum*, 3 were *C. burmanii*, 1 was *C. burmanii*/*cassia*/*loureiroi*, 1 was *C. aromaticum*, and 9 were labelled as cassia; no information on botanical variety was provided for the remaining 29). The remaining 34 samples were commercialized as bark (17 *C. verum* and 2 cassia; no information on botanical variety was provided for the remaining 15). All test samples were kept in proper sealing bags at room temperature to avoid humidity effects. For those cinnamon samples that were purchased in sticks, milling was used to produce powder for testing.

To assist the interpretation of the cinnamon spectra, reference standards for cinnamaldehyde (≥99%), eugenol (≥99%), coumarin (99%), and cinnamyl acetate (≥97%), representing the most abundant chemical compounds in cinnamon essential oil, were also measured.

### 2.2. Cinnamon Admixtures

To determine the content of cassia in Ceylon, ground cinnamon admixtures were prepared by spiking a *Ceylon cinnamon* matrix with increasing concentrations of a cassia cinnamon matrix. The spiking levels considered (in % *w*/*w* cassia) were as follows: 0 (pure Ceylon), 5, 9, 20, 30, 50, 70, and 100 (pure cassia). The *Ceylon cinnamon* matrix used in the admixtures was prepared by mixing equal amounts of three Ceylon samples, while the cassia cinnamon matrix was prepared in the same manner using three cassia samples.

### 2.3. Raman Measurements and Spectral Processing

Raman measurements on cinnamon samples, cinnamon mixtures, and reference standards were performed using a benchtop Vertex 70 Fourier Transform (FT) spectrometer (Bruker, Kontich, Belgium) equipped with a RAM II module in backscattering mode. An air-cooled diode-pumped Nd:YAG laser operating at 1064 nm was used for excitation to avoid excess fluorescence, and a liquid N_2_-cooled ultrahigh sensitivity Ge detector was used for the detection of the Raman signal. The excitation laser was kept at 150 mW during the measurements to avoid potential sample damage. The spectra were collected with a Raman shift scanning range of 200–3200 cm^−1^, a resolution of 4 cm^−1^, and 250 scans that were averaged for each spectrum, with the v.7.5. (2014) OPUS software (Optik Instruments, Brno-Medlánky, Czech Republic) used for data acquisition.

For the Raman measurements, samples were deposited into glass vials, and no further sample preparation was performed. The glass vials were subsequently placed on the RAMII sample holder, and the spectra were recorded through the side of the vial, after focusing the laser to attain a sufficient Raman signal. The sample holder was kept fixed for all measurements, and no interference from the glass vials was observed. Each sample was measured in triplicate, and the average spectrum was used for further analysis. As cinnamon samples and mixtures were in powder form, each glass vial was shaken before each replicate measurement to ensure homogeneity and a new measurement point.

Raman spectra were subjected to multivariate statistical analysis using the software package SIMCA 18 (Umetrics, Umeå, Sweden). Prior to any statistical analysis, spectral pre-processing was carried out by standard normal variate (SNV), which is a normalization process that allows spectral correction by centering the midpoint of each spectrum and standardizing it with the overall variance. PCA is an unsupervised method that reduces the dimensionality of the original data and was applied for differentiation among the spectra.

This study aimed to develop a rapid method to detect potential substitution in ground cinnamon products on the European market. Due to the high risk of adulteration—especially in products labelled as *Ceylon cinnamon*—and the lack of samples with confirmed identities, supervised classification methods were not suitable. Therefore, an untargeted Raman spectroscopy approach was used.

The results were validated with complementary techniques, including GC-MS and XRF, to ensure robustness and reliability. XRF was used as a targeted screening method, showing that the elemental content of cassia samples is generally lower than that of *Ceylon cinnamon*, and as such, the elemental profile determined by XRF was used to detect substitution of *Ceylon cinnamon* by cassia. The detection of certain elements (e.g., Al, Si, Ti, Cr, Fe, Zr, and Pb) was also used as an indication of substitution with organic matter and/or effect by the material used to mill the cinnamon sticks. GC-MS was based on the analysis of several volatile compounds (e.g., camphor, cinnamaldehyde, eugenol, coumarin, cinnamyl acetate) for the detection of substitution of *Ceylon cinnamon* with cassia as well as the substitution of bark with other parts of the cinnamon plant (leaves, flowers, roots, seeds), based on the difference in relative abundances of the selected compounds [20,21].

## 3. Results

### 3.1. Raman Characterization of Cinnamon Species

**Figure 1:**
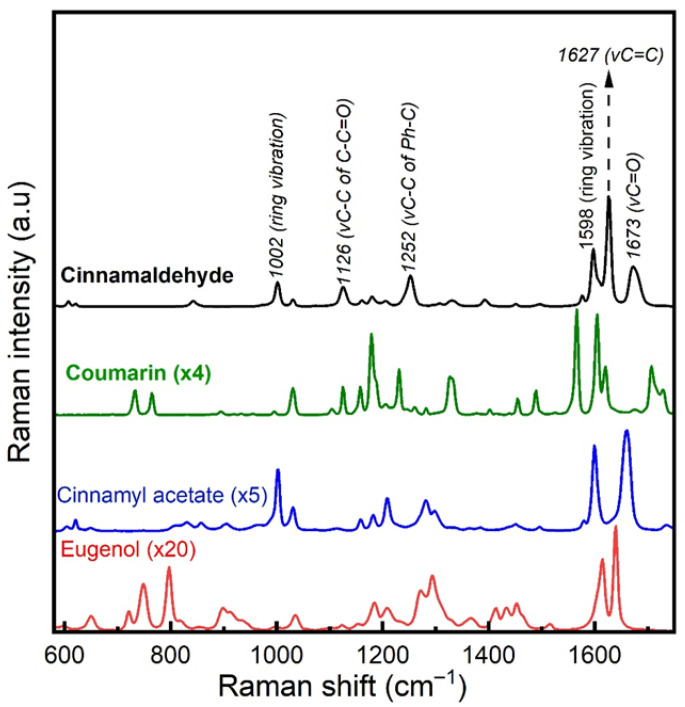
Raman spectra of the most abundant chemical compounds present in cinnamon essential oil.

Prior to the measurements of the cinnamon samples, reference spectra of the most abundant chemical compounds present in the essential oil were recorded to assist the interpretation of the cinnamon spectra. Figure 1 shows the Raman spectra of these substances and the assignment for some of the main bands observed. Since the overall Raman response of cinnamaldehyde is significantly stronger than the other compounds under the same measurement conditions, the spectral intensities of coumarin, cinnamyl acetate, and eugenol were scaled (multiplied by a factor of 4, 5 and 20 respectively) for better visualization and comparison.

The spectral profiles of these compounds exhibit Raman band overlapping across the fingerprint region (600–1800 cm^−1^), but with plausible relative intensity variations. Cinnamaldehyde appears to be a very strong Raman scatterer, due to the conjugated nature of cinnamaldehyde, which contains several double bonds, resulting in a high Raman scattering cross section [22]. This finding, combined with the fact that it is the most abundant volatile found in cinnamon bark, indicates that cinnamaldehyde is expected to show the strongest scattering contribution in the Raman spectra of cinnamon among the aromatic compounds of the essential oil, even though its total concentration in ground cinnamon does not exceed 10% [23].

**Figure 2:**
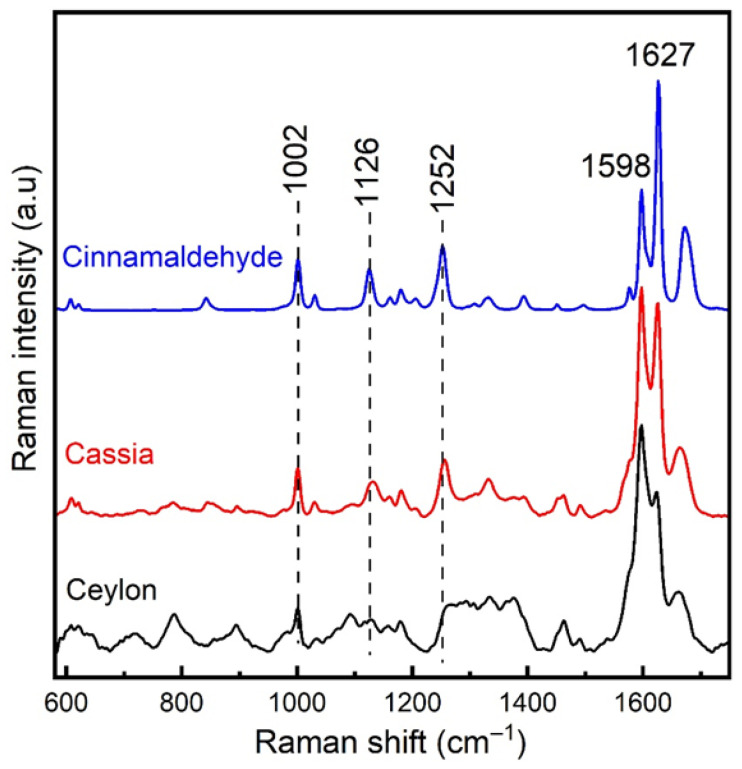
Raman spectra of cassia- and Ceylon-labelled cinnamon samples together with the reference spectrum of pure cinnamaldehyde for comparison. Spectra are normalized to their most intense peak, and an offset was applied for comparison.

Considering the above, Figure 2 shows the Raman spectra of cassia and Ceylon-labelled cinnamon samples together with the reference spectrum of pure cinnamaldehyde for comparison. The graph demonstrates observable similarities between cassia cinnamon and cinnamaldehyde spectra, whereas such similarities are less evident for Ceylon and cinnamaldehyde. It is interesting to note that the 1598/1627 cm^−1^ band ratio values in both cassia and *Ceylon cinnamon* are somewhat different from the corresponding intensity ratio in pure cinnamaldehyde, since additional spectral contribution from other aldehydes (e.g., coniferyl aldehyde) present in the lignin part of cinnamon bark is also expected at this frequency region, albeit less prominent [24]. However, upon comparison of these intensity ratio values, cassia shows much better correlation with pure cinnamaldehyde compared to Ceylon.

In addition to the above, cinnamaldehyde displays some characteristic Raman bands at 1002, 1126, and 1252 cm^−1^, which can be clearly seen in the spectrum of cassia cinnamon, indicating that the presence of cinnamaldehyde is more prominent in this species, in agreement with the findings of previous works [25,26]. Moreover, other lignin-related Raman features are also present in this frequency range, providing significant spectral contribution to the Raman signal of Ceylon and cassia cinnamon [24].

According to Figure 1, coumarin also exhibits strong Raman scattering, and it comprises multiple spectral bands overlapping with Raman bands of cinnamaldehyde, across the 1000–1700 cm^−1^ range. Hence, in addition to cinnamaldehyde, scattering from coumarin may also contribute to the overall Raman spectrum of cassia, although weakly, since the maximum concentration of coumarin in cinnamon powder does not exceed 1% [23]. Similar spectral contribution is not expected to be seen for Ceylon, due to its significantly lower content of coumarin than cassia cinnamon [23]. Further Raman activity could also arise from cinnamic acid, which is the most abundant free phenolic acid in cinnamon, found at relatively higher concentrations in cassia compared to Ceylon [27], due to the presence of several characteristic Raman bands over the 1000–1700 cm^−1^ range that overlap with spectral features of cinnamaldehyde and coumarin [28]. However, estimations of cinnamic acid concentration in cinnamon powder are reported to be much less than 1% [27], which implies that a relevant contribution to the spectrum of cassia is expected to be very weak.

An additional source of vibrational contribution below 1500 cm^−1^ in both cassia and Ceylon spectra contains scattering from cellulose/hemicellulose structures that are generally present in cinnamon bark [24]. Overall, the observed Raman spectra of both cinnamon species result from the superimposition of vibrational bands ascribed to the ligno-cellulosic matrix, the major aromatic compounds (cinnamaldehyde, coumarin), and phenolic acids, albeit at varying intensities depending on the type of cinnamon species.

### 3.2. Chemometric Analysis and Data Interpretation

#### 3.2.1. Identification of Types of Cinnamon Substitution

**Figure 3:**
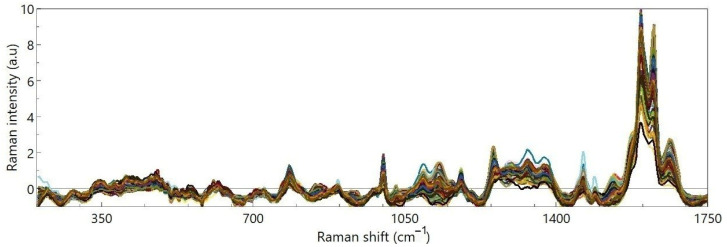
Overview of the pre-processed Raman spectra taken on ground cinnamon samples.

Figure 3 shows an overview of the pre-processed FT-Raman spectra (unprocessed spectra are provided in Figure S1) recorded for all the ground cinnamon samples investigated in this study.

**Figure 4:**
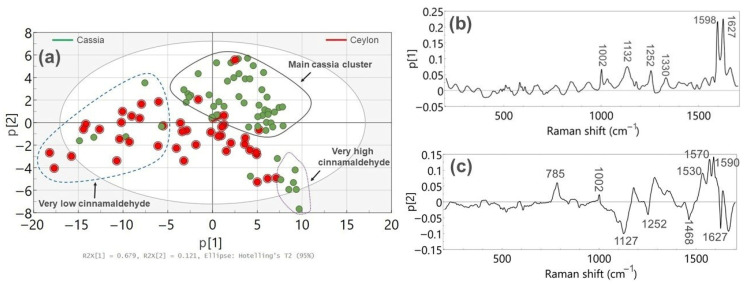
(**a**). PCA scores plot (p2 vs. p1) of the cinnamon samples. Cassia cinnamon samples are depicted in green, while Ceylon-labelled samples are shown in red. (**b**). Loading plot for p1. (**c**). Loading plot for p2. The ellipse in 4a represents a single Hotelling’s T^2^ ellipse at a 95% confidence level.

After applying SNV to the spectra presented above, PCA was employed to explore differences among samples. Figure 4a shows an overview of the PCA investigation for the cinnamon samples, where the first two principal components (p1 and p2) explain approximately 80% of the total variation.

The PCA scores plot shows that the *Ceylon cinnamon*-labelled samples (shown in red) appear highly spread along the first principal component p1. The left part of the scores plot is mainly characterized by Ceylon-labelled samples that exhibit a very weak cinnamaldehyde Raman signal (shown in blue dashed circle), whereas the central part denotes Ceylon specimens with a higher cinnamaldehyde signal. In contrast, cassia cinnamon samples are largely projected at the upper central/right part of the scores plot, where cinnamaldehyde scattering becomes more prominent (shown in black circle), while a small subset of cassia samples exhibiting a predominant cinnamaldehyde Raman signal is found at the bottom right part of the score plot (shown in dotted purple circle). To further relate the PCA clustering to differences in chemical composition, the loading plots of the principal components were examined, as illustrated in Figure 4b,c.

Figure 4b shows that the first principal component (p1) explains 68% of the total spectral variance and is mainly influenced by Raman features in the 1000–1700 cm^−1^ region. The strongest contributions arise from the bands centred at 1598 and 1627 cm^−1^. These bands are primarily associated with aromatic ring and C=C stretching vibrations of cinnamaldehyde, which is the dominant component of cinnamon essential oil. Additional contributions to these bands may originate from other aromatic constituents such as coniferyl aldehyde, coumarin, and, to a lesser extent, cinnamic acid [28].

Weaker Raman bands in the 1000–1500 cm^−1^ spectral region also contribute to p1 and are mainly associated with lignocellulosic components such as cellulose and (hemi)cellulose. Overall, the loading profile indicates that variations in the intensity of cinnamaldehyde-related Raman bands represent the dominant factor governing the separation of samples along p1. Consequently, the systematic shift observed in the PCA score plot (Figure 4a) from left to right reflects an increasing contribution of cinnamaldehyde in the analyzed samples.

The Ceylon-labelled samples exhibiting very low Raman cinnamaldehyde signal at the left cluster of Figure 4a are also characterized by increased amounts of either camphor (shown by GC-MS analysis) and/or some mineral elements such as Al, Ti, Si, Fe, Ni, and Zr (shown by XRF analysis), which could indicate partial or total substitution by either *Cinnamon camphora* (rich in camphor and poor in cinnamaldehyde) and/or inorganic matter rich in such mineral elements, respectively [20,21]. A contributing factor to the mineral presence could also stem from the material used to mill the cinnamon sticks to produce the powder [20]. For the presence of camphor, an alternative scenario could entail partial or total substitution of the cinnamon bark by the root of the tree, which also contains camphor in substantial amounts [29]. The above scenarios would explain the low Raman signal of cinnamaldehyde seen in these spectra, suggesting that these samples are suspicious. Unfortunately, it was not possible to independently test these two possibilities because certified *Cinnamon camphora* or root samples were not readily available, and access to botanically verified material would require collaboration with specialized botanical gardens or field collection.

In addition to these Ceylon-labelled samples, a few cassia samples are also projected within this cluster, which contrasts with the rest of the cassia cinnamon. According to the XRF results, relatively high concentrations of the same mineral elements were also observed for these samples due to potential substitution by inorganic matter, indicating that these cassia specimens are also suspicious [20,21]. Overall, all samples located in this region of the scores plot were consistently found suspicious by the two confirmatory techniques, which further validates the Raman findings. Hence, the absent/low cinnamaldehyde Raman signal is a critical factor for detecting the substitution of either pure Ceylon or cassia cinnamon with *Cinnamon camphora*, other parts of the plant (e.g., the root), and/or inorganic matter. Raman spectroscopy is thus an efficient method to detect this type of substitution due to the strong Raman scattering cross section of cinnamaldehyde, which allows the monitoring of its content variations even at low concentrations.

In contrast to Raman spectroscopy, infrared spectra (IR) of cinnamon are mainly dominated by the major chemical compounds such as lignin and (hemi)cellulose, while the signal assigned to cinnamaldehyde is relatively weak and broadly overlaps with the peaks of the major components. To further demonstrate the difference in strength between Raman and IR activities of cinnamaldehyde, both types of spectra were recorded for a cassia cinnamon sample before and after removal of the essential oil, where, in the latter case, the aromatic compounds such as cinnamaldehyde are eliminated. The comparison can be seen in Figures S2 and S3, demonstrating the advantage of using Raman spectroscopy over IR in probing cinnamaldehyde.

Moreover, the Ceylon-labelled samples located in the central-right part of Figure 4a are separated by the main cluster of cassia samples (shown in a black circle) across both p1 and p2, although the separation is more evident in p2. As a result, these Ceylon-labelled samples do not raise any suspicion of obvious substitution. Most of the samples within the cassia cinnamon cluster appear slightly shifted to the right compared to the majority of the Ceylon-labelled samples located in the centre of p1, due to the higher cinnamaldehyde Raman signal detected. This can be related to higher cinnamaldehyde content present in cassia species, as already shown in other works [25,26,30]. Although this variation in cinnamaldehyde content is evident, it is not strong enough to yield a clear distinction based on p1 alone, as previously shown [31].

A more notable separation between the two species is detected across p2, where spectral contributions along the 1000–1500 cm^−1^ frequency region are more prominent, as shown in Figure 4c. In this range, the relative intensities of overlapping Raman bands originating from cinnamaldehyde and coumarin are more comparable, and they also exhibit additional overlapping with Raman features of lignin, (hemi) cellulose, and cinnamic acid [24,28]. As a result, the different relative intensities attributed to these compounds found also at different concentrations could trigger the observed variations between cassia and Ceylon species along p2. Finally, other phenolic acids, such as p-coumaric and ferulic acids derived from cinnamic acid, which are generally found at higher concentrations in cassia cinnamon than in Ceylon, could also enhance the overall Raman signal in this range, although it is less likely due to their very low concentration in cinnamon. In this respect, the few Ceylon-labelled samples that appear within the cassia cluster indicate potential substitution, which is in agreement with the GC-MS results [20,21].

**Figure 5:**
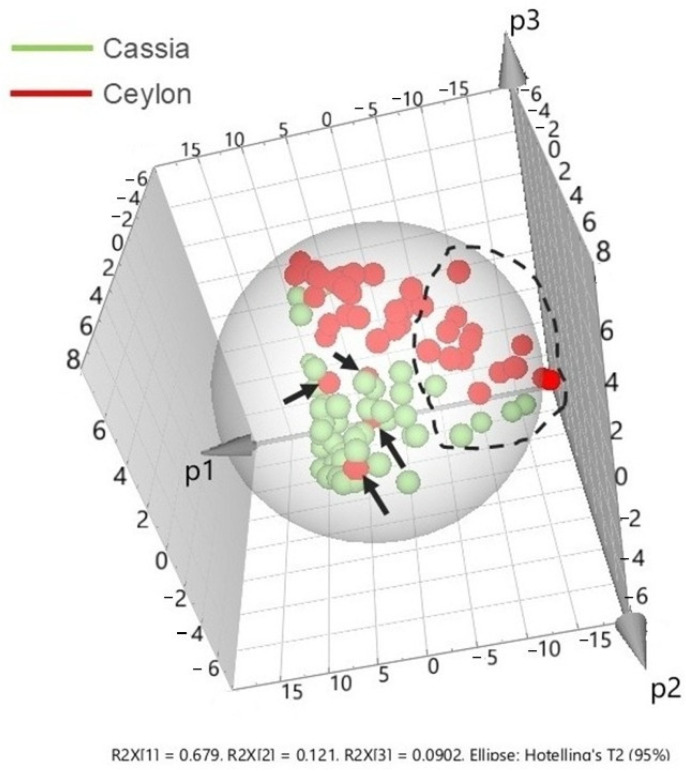
3D PCA scores plot of all cinnamon samples. Cassia cinnamon samples are depicted in green, while Ceylon-labelled samples are shown in red. Black arrows show the Ceylon-labelled samples substituted by cassia, while the black dashed circle represents the samples with low cinnamaldehyde Raman signal, denoting substitution with *Cinnamon camphora*/root or inorganic matter. The ellipse represents a single Hotelling’s T^2^ ellipse at a 95% confidence level.

To shed more light on these findings, the third principal component (p3) is assessed in a 3D plot shown in Figure 5. The right part of the p1 in the scores plot is mainly ascribed to Ceylon-labelled samples that exhibit a very weak cinnamaldehyde Raman signal (black dashed circle), whereas moving towards the centre, the presence of cinnamaldehyde becomes more prominent. On the other hand, cassia cinnamon samples are now projected at the centre and bottom left part, where cinnamaldehyde is systematically increasing.

In this PCA plot, the Ceylon and cassia samples located at the left of the scores plot are suspicious of substantial substitution with either *Cinnamon camphora* or cinnamon root and/or inorganic matter, as already demonstrated in Figure 4a. The rest of the Ceylon-labelled samples that do not raise obvious suspicions are now located at the upper central part of the plot and separated from the main cassia cluster along p3. Finally, the four Ceylon-labelled samples that are clearly found within the cassia cluster in p3 (pointed by black arrows) denote significant substitution, in line with the observations in Figure 4a and confirmed by GC-MS findings [20,21].

Overall, these results demonstrate that PCA primarily reflects variations in the relative abundance of key aromatic constituents of cinnamon, particularly cinnamaldehyde and related phenylpropanoids, as well as major constituents such as lignin and (hemi)cellulose. Considering the total number of Ceylon-labelled samples (*n* = 48, Table S1), approximately 37% of the specimens are identified as suspicious based on the combined Raman, GC-MS, and XRF results, which represents a substantial fraction of the dataset, and the consistency across multiple analytical techniques supports the robustness of the observed trends and the validity of the proposed approach.

#### 3.2.2. Semi-Quantitative Evaluation of Cassia in *Ceylon cinnamon*

**Figure 6:**
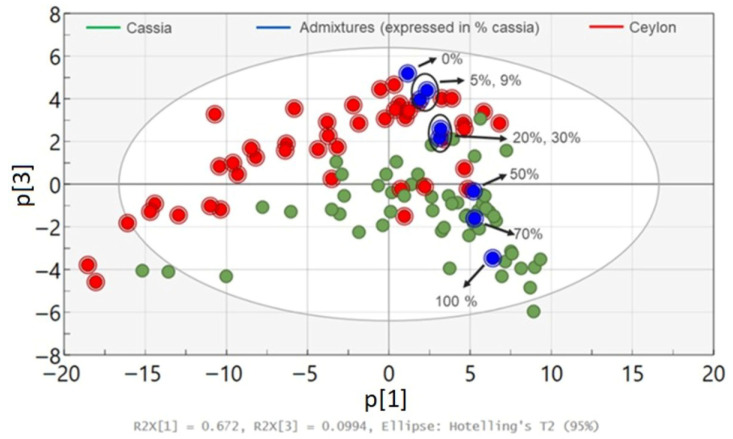
PCA scores plot (p3 vs. p1) of all cinnamon samples and admixtures. The ellipse represents a single Hotelling’s T^2^ ellipse at a 95% confidence level.

To further elaborate on the qualitative assessment of Ceylon substitution by cassia, a semi-quantitative approach was employed by reconstructing the PCA plot using prepared admixtures of Ceylon and cassia cinnamon matrices at various relative concentrations, as seen in Figure 6. It is noted that three replicate measurements were performed for each admixture, and the average spectrum was subsequently calculated and used for PCA. In addition, the choice of the individual Ceylon and cassia samples that were mixed to form the matrices was based on the PCA findings in Figure 4a, also supported by GC-MS performed on these specimens [20].

From the PCA plot, the scores corresponding to the admixtures (shown in blue and expressed as % of cassia) are homogeneously spread across the third principal component from the Ceylon region found at the centre of the plot towards the right part where the cassia region is located. It is also observed that between 5 and 9%, as well as between 20 and 30% cassia concentrations, the relevant scores appear very close to each other due to minor spectral variations detected.

Figure 6 shows that the 5% and 9% admixtures fall within the Ceylon cluster, indicating that the detection of cassia below 10% in *Ceylon cinnamon* was not feasible using the present approach. The 20% and 30% admixtures begin to deviate from the Ceylon cluster and move towards the cassia region, although they remain relatively close to the Ceylon samples and are clearly separated from the main cassia cluster. In contrast, the 50% and 70% admixtures are located within the cassia cluster, indicating a clear shift in the spectral profile at higher cassia proportions. Based on these observations, the presence of cassia in *Ceylon cinnamon* appears to become detectable at around 20% cassia.

However, the 20% and 30% mixtures still show partial overlap with a small number of Ceylon- and cassia-labelled samples, suggesting that this level should be considered an approximate detection threshold rather than a definitive limit of detection. Further analysis with a larger dataset would be required to confirm the robustness of this threshold. The limited overlap observed may also reflect the natural variability expected in commercial cinnamon samples obtained from the market, such as those investigated in this study. This provides more evidence that the current method could be a more realistic way to study ground cinnamon products commonly available to consumers.

## 4. Discussion

Based on the results shown in Figure 4a and Figure 5, it is evident that FT-Raman combined with PCA provides a very efficient and fast approach to detect the substitution of Ceylon and cassia species by *Cinnamon camphora*, other parts of the plant (e.g., root), and/or inorganic matter, using only cinnamaldehyde as the main marker along p1. While complementary techniques such as GC-MS and XRF were considered to confirm the type of substitution, FT-Raman spectroscopy alone appears to be a reliable method to flag suspicious samples. This was demonstrated by the low Raman activity observed at 1627 cm^−1^ relative to 1598 cm^−1^, which is opposite to the intensity ratio exhibited in pure cinnamaldehyde. Furthermore, other cinnamaldehyde-related Raman bands at 1002, 1126, and 1252 cm^−1^ are too weak to be seen in suspicious cinnamon and possibly overlap with other Raman features assigned to the lignocellulosic matrix. As a result, the FT-Raman spectra of these samples can directly show the significantly lower cinnamaldehyde content compared to the rest of the samples. This is the first time that this type of cinnamon substitution has been explored using vibrational spectroscopy, which constitutes a significant novelty in the field of cinnamon authenticity.

Regarding the substitution of Ceylon by cassia, inspection of the spectral differences observed along p1, p2, and p3 components is used. Besides the observations for cinnamaldehyde along p1, the FT-Raman results displayed separation between the two species across p2, where spectral contributions along the 1000–1500 cm^−1^ frequency region are more prominent. In this range, the relative intensities of overlapping Raman bands of cinnamaldehyde and coumarin are more comparable, and additional overlapping with Raman features of the lignocellulosic matrix and cinnamic acid is also present. Thus, the different relative intensities attributed to these compounds at different concentrations could trigger the observed variations between cassia and Ceylon samples along p2. In addition, the Raman signal from p-coumaric and ferulic acids derived from cinnamic acid at higher concentrations in cassia specimens than in Ceylon could also enhance the overall Raman signal, although less likely due to their very low concentration in cinnamon.

The present FT-Raman method demonstrates the possibility to discriminate the two cinnamon species directly in ground form, with no further preparation needed, in contrast to the SERS study conducted by Nelson et al. [19], where the authors performed screening of cinnamon authenticity using the essential oil. For the latter, sample preparation is required to extract the essential oil from the cinnamon samples, as well as for the nanoparticle substrates that were used for the SERS experiment. Furthermore, the FT-Raman method used three principal components explaining almost 90% of the variance, which is in good agreement with the FTIR methods developed by Lopes et al. [14] and Li et al. [12], where they used the same number of principal components, describing approximately 95% of the total variance. Although the work by Li et al. was not performed directly on cinnamon samples (instead, liquid films containing cinnamon oil were used) [12], the feasibility of employing both Raman and FTIR techniques in studying cinnamon speciation highlights the full potential that vibrational spectroscopy coupled to multivariate statistics possesses for fast and efficient screening.

Moreover, Figure 6 shows that the lowest concentration of cassia in Ceylon that can be detected by our FT-Raman method is estimated to be around 20%, which is higher than previously reported values based on NIR [15,16,17] and MIR data [15]. It should be noted that direct comparison of our detection estimation with the corresponding values given in the above studies is not straightforward, because these values were determined using IR spectroscopy (instead of Raman spectroscopy used in the present work) and different statistical tools, such as Partial Least Squares-Discriminant Analysis (PLS-DA) [16,17] and PLS-Regression [15].

In another study led by Lixourgioti et al., FTIR combined with PLS-DA showed that the level of detection for a mixture of cassia and cinnamon spent in Ceylon had to be 20% to achieve reliable classification results. Although this value is in line with our observations, the authors not only used PLS-DA for their investigation, but also performed quantification of two adulterants in Ceylon, in contrast to the present work that considered only one adulterant (cassia) [13]. Furthermore, size homogenization of their ground samples was performed before the measurements, whereas no prior sample preparation was carried out in the current work.

In comparison to chromatography, where targeted analysis is applied for the determination of cinnamon authenticity (e.g., coumarin, cinnamaldehyde) [12,23], vibrational spectroscopy relies on the untargeted analysis of a set of cinnamon samples to discriminate differences among them, which is very useful for the routine analysis of unknown specimens usually performed by control laboratories. Moreover, the developed Raman method is much faster (single measurement takes approximately ~15 min), with no sample preparation needed, whereas spectral acquisition for the chromatographic methods takes more time on average (~60 min), including the sample preparation step [13].

## 5. Conclusions

This study presents the development of a novel untargeted FT-Raman method combined with multivariate statistics as an analytical tool to identify suspicious ground cinnamon samples, without any prior sample preparation. This is the first systematic investigation of a large number of commercially available cinnamon samples (>100) from across the European market that ensures a level of variability representative of real-world conditions encountered in control laboratories.

A key innovation is that FT-Raman spectroscopy can detect suspicious Ceylon and cassia species based on a weak (or absent) cinnamaldehyde signal, which is indicative of potential substitution with *Cinnamon camphora*, other parts of the plant (e.g., the root), and/or inorganic matter, as confirmed by GC-MS and XRF analyses. To our knowledge, this is the first study reporting this type of substitution by means of vibrational spectroscopy, outlining the advantage of Raman over infrared spectroscopy, due to the strong Raman scattering cross-section of cinnamaldehyde enabling its efficient detection.

In addition, the developed method allows differentiation between cassia and *Ceylon cinnamon*, with a detection level of ~20% cassia in *Ceylon cinnamon*. Although this sensitivity is lower than that reported for some infrared-based methods, the proposed approach offers significant advantages in terms of speed, simplicity, and minimal sample preparation.

Overall, the described FT-Raman approach demonstrates strong potential as an efficient and rapid method for identifying suspicious cinnamon samples, with broader implications in the fields of food science as well as health and consumer protection.

Future work should focus on validating the screening capability of this method using a larger set of cinnamon samples with certified purity (especially for Ceylon, which is mainly prone to fraud), as well as samples of *Cinnamon camphora* and/or cinnamon root that were not available in the present study, using supervised techniques (e.g., PLS-DA). Additionally, exploring the transferability of this method to handheld Raman devices and its use in conjunction with other complementary NIR/FTIR methods would provide a more holistic assessment of the various types of cinnamon substitution and enhance the efficiency of detecting suspicious samples.

## Data Availability

The original contributions presented in the study are included in the article/Supplementary Materials, further inquiries can be directed to the corresponding author.

## Supplementary Materials

The following supporting information can be downloaded at https://www.mdpi.com/article/10.3390/foods15081311/s1: Table S1: Summary of information about the test samples analyzed; Figure S1: Overview of the unprocessed Raman spectra taken on ground cinnamon samples; Figure S2: IR spectra of pure cinnamaldehyde and untreated and treated cassia cinnamon; Figure S3: Raman spectra of pure cinnamaldehyde and untreated and treated cassia cinnamon.
