# Supplementary material for: Development of a New FT-Raman Method for the Investigation of Cinnamon Authenticity
Source: Foods. 2026 Apr 10;15(8):1311. doi: 10.3390/foods15081311 (PMC13114356; doi:10.3390/foods15081311)
Supplement: Supplementary file 1 [file foods-15-01311-s001.zip › foods-4202559-supplementary.pdf]

foods

MDPI

Supplementary Information

Development of a new FT-Raman method for the investigation of cinnamon authenticity

Konstantinos I. Chatzipanagis *, Ana Boix Sanfeliu

European Commission, Directorate-General Joint Research Centre, Directorate-F Health and Food, Retieseweg 111, Geel 2440, Belgium.ffiliation 1; e-mail@e-mail.com

* Correspondence: kchatzipanagis@gmail.com;

S1. Overview of cinnamon samples used for analysis

Table S1: Summary of the test samples analyzed.

It is noted that samples Cin087, Cin 088, Cin 089, Cin 090, Cin 091 and Cin 092 are not included in table S1 and were not analyzed because they were purchased as leaves and as a result, authenticity assessment was not possible.

Sample id	Label variety	BIO/ NON BIO	Purchase form	Origin
Cin001	?	NON BIO	Ground	?
Cin002	?	NON BIO	Ground	?
Cin003	?	NON BIO	Ground	?
Cin004	?	NON BIO	Ground	?
Cin005	?	BIO	Ground	?
Cin006	?	NON BIO	Ground	?
Cin007	?	NON BIO	Ground	?
Cin008	?	NON BIO	Ground	?
Cin009	?	NON BIO	Ground	?

Academic Editor: Firstname Lastname

Received: date
Revised: date
Accepted: date
Published: date

Copyright: © 2026 by the authors. Submitted for possible open access publication under the terms and conditions of the Creative Commons Attribution (CC BY) license.

Cin010	?	NON BIO	Ground	?
Cin011	Ceylon	BIO	Ground	Madagascar
Cin012	Ceylon	BIO	Ground	Sri Lanka
Cin013	?	BIO	Ground	NON EU
Cin014	Ceylon	BIO	Ground	Madagascar
Cin015	Burma nni	BIO	Ground	?
Cin016	Aroma ticum	BIO	Ground	?
Cin017	?	NON BIO	Ground	Ceylan and Indonesia
Cin018	Ceylon	BIO	Ground	NON EU
Cin019	Ceylon	BIO	Ground	NON EU
Cin020	Ceylon	BIO	Ground	NON EU
Cin021	Ceylon	NON BIO	Ground	?
Cin022	Ceylon	NON BIO	Ground	?
Cin023	Cassia	NON BIO	Ground	
Cin024	Cassia	BIO	Ground	Vietnam
Cin025	Ceylon	NON BIO	Ground	?
Cin026	?	NON BIO	Ground	Vietnam
Cin027	Burma nni	NON BIO	Ground	?
Cin028	Burma nni	BIO	Ground	?
Cin029	Ceylon	NON BIO	Ground	?
Cin029 batch 2	Ceylon	NON BIO	Ground	?
Cin030	Cassia	NON BIO	Ground	?
Cin030 batch 2	Cassia	NON BIO	Ground	?

Cin031	?	NON BIO	Ground	Sri Lanka
Cin032	Ceylon	NON BIO	Stick	Sri Lanka
Cin033	Ceylon	NON BIO	Stick	Sri Lanka
Cin034	Ceylon	NON BIO	Stick	Sri Lanka
Cin035	Ceylon	BIO	Stick	Sri Lanka
Cin036	Ceylon	BIO	Stick	Sri Lanka
Cin037	Ceylon	BIO	Stick	?
Cin039	Ceylon	NON BIO	Stick	Sri Lanka
Cin040	Ceylon	BIO	Ground	?
Cin041	Ceylon	NON BIO	Ground	Sri Lanka
Cin042	Ceylon	NON BIO	Stick	Sri Lanka
Cin043	Ceylon	BIO	Ground	?
Cin044	Ceylon	NON BIO	Ground	Madagascar
Cin045	Ceylon	NON BIO	Ground	Sri Lanka
Cin046	Ceylon	BIO	Ground	India
Cin047	Ceylon	BIO	Stick	?
Cin048	Ceylon	BIO	Stick	Sri Lanka
Cin049	Ceylon	BIO	Ground	?
Cin050	Ceylon	NON BIO	Ground	Sri Lanka
Cin051	Ceylon	NON BIO	Ground	Sri Lanka
Cin052	Cassia	NON BIO	Stick	Indonesia
Cin053	?	NON BIO	Ground	Ceylan and Indonesia
Cin054	Ceylon	NON BIO	Stick	?
Cin055	?	?	Ground	?

Cin056	?	NON BIO	Stick	Indonesia
Cin057	Ceylon	BIO	Ground	Sri Lanka
Cin058	Ceylon	BIO	Ground	Sri Lanka
Cin059	Ceylon	BIO	Stick	Sri Lanka
Cin060	?	NON BIO	Ground	?
Cin061	?	NON BIO	Stick	?
Cin062	?	NON BIO	Stick	?
Cin063	?	NON BIO	Ground	Tropics
Cin064	burmanni/ cassia/ loureiroi	NON BIO	Ground	?
Cin065	Ceylon	BIO	Ground	Sri Lanka
Cin066	?	NON BIO	Stick	Vietnam
Cin067	?	NON BIO	Ground	Vietnam
Cin068	?	NON BIO	Ground	Outside EU
Cin069	Ceylon	BIO	Stick	Outside EU
Cin070	Cassia	BIO	Ground	Vietnam
Cin071	Ceylon	BIO	Ground	Outside EU
Cin072	Cassia	?	Ground	?
Cin073	Ceylon	?	Ground	?
Cin074	Cassia	BIO	Ground	India
Cin075	Ceylon	BIO	Ground	Madagascar
Cin076	Ceylon	BIO	Ground	Sri Lanka
Cin077	Ceylon	BIO	Stick	Sri Lanka
Cin078	?	NON BIO	Stick	Indonesia
Cin079	?	NON BIO	Stick	?

Cin080	?	NON BIO	Ground	?
Cin081	?	NON BIO	Stick	?
Cin082	?	NON BIO	Ground	?
Cin083	Cassia	NON BIO	Ground	?
Cin084	?	NON BIO	Stick	Vietnam
Cin085	?	NON BIO	Ground	Vietnam
Cin086	?	NON BIO	Ground	?
Cin093	?	NON BIO	Stick	?
Cin094	?	NON BIO	Ground	?
Cin095	?	NON BIO	Stick	?
Cin096	?	NON BIO	Ground	?
Cin097	?	NON BIO	Stick	China
Cin098	Cassia	NON BIO	Ground	?
Cin099	Cassia	NON BIO	Stick	?
Cin100	Ceylon	NON BIO	Stick	?
Cin101	?	NON BIO	Stick	?
Cin102	?	NON BIO	Ground	?
Cin 103	?	NON BIO	Stick	Sri Lanka

Cin 104	?	NON BIO	Stick	Sri Lanka
Cin 105	?	NON BIO	Stick	Madagascar
Cin 106	?	NON BIO	Ground	Madagascar

S2. Unprocessed FT-Raman spectra of all ground cinnamon samples

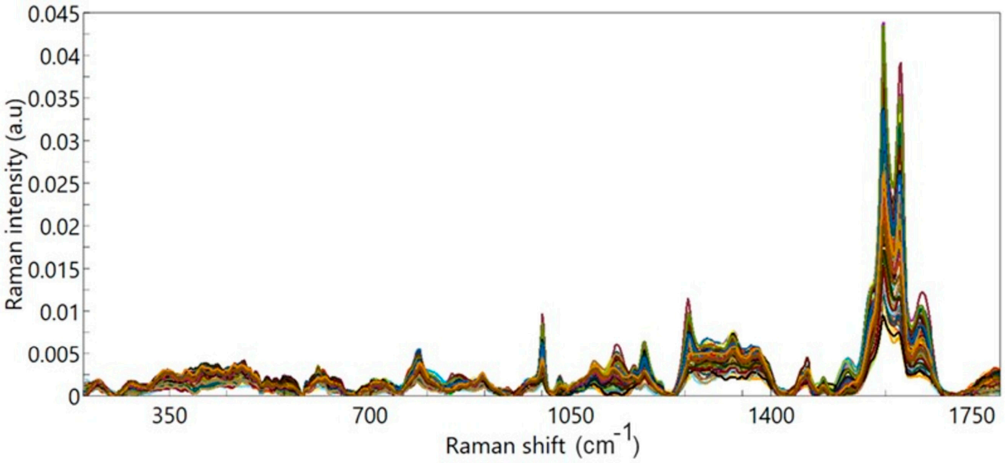

Figure. S1. Overview of the unprocessed Raman spectra taken on ground cinnamon samples.

S3. Raman and IR spectroscopy on cassia cinnamon before and after removal of the essential oil

30 g of cassia cinnamon bark was broken into small pieces using a mortar and some of these pieces were subsequently grounded (untreated). The remaining pieces were immersed into 150 ml distilled water and heated at 100°C for approximately 3 hours to remove the essential oil containing the aromatic compounds. After the removal of the essential oil, the pieces were dried and grounded as well (treated). Raman and infrared (IR) spectra of the untreated and treated cinnamon powder were recorded to investigate the spectral changes attributed to the presence of aromatic compounds. The IR (Figure S2) and Raman (Figure S3) spectra of the untreated and treated cinnamon powder are plotted together with the corresponding spectra of pure cinnamaldehyde, the latter being the most abundant component among all aromatic compounds present in cinnamon. The IR cinnamon spectra and the Raman spectra of pure cinnamaldehyde were normalized to unity and an offset was arbitrarily applied in both plots for better visualization and comparison.

Fig. S2 shows that the IR spectra of the treated and untreated cinnamon remain largely unaffected, with only minor differences observed over the frequency range. These spectral changes are indicated by the dashed arrows, and they coincide with the most notable IR bands of pure cinnamaldehyde. On one hand, although the frequency range above 900 cm^{-1} exhibits severe band overlapping due to multiple IR features, an intensity decrease in the band shoulder at ~ 1667 cm^{-1} is observed for the treated cinnamon, which coincides

with the strongest absorption band of cinnamaldehyde. On the other hand, the two IR bands at 686 and 745 cm^{-1} can be better visualized in the untreated cinnamon, whereas their corresponding intensities in the treated sample are significantly decreased. Overall, the cinnamaldehyde related IR bands are weakly present in the cinnamon spectra and substantially overlap with the signal originating from the major cinnamon compounds (lignin, cellulose), demonstrating that IR spectroscopy is sensitive to the relative concentrations of the various compounds found in cinnamon.

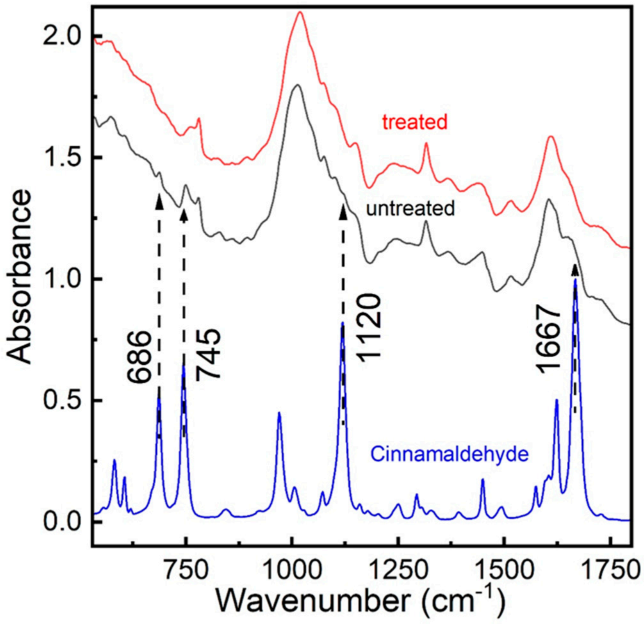

Figure. S2. IR spectra of pure cinnamaldehyde, untreated and treated cassia cinnamon.

Figure S3 shows the corresponding Raman spectra of the treated and untreated cinnamon, where significant variations are observed over the fingerprint region. Minor bands below 1000 cm^{-1} and the more pronounced features at 1000, 1127 and 1254 cm^{-1} assigned to cinnamaldehyde are clearly observed in the Raman spectrum of untreated cinnamon, while they almost disappear in the spectrum of the treated sample. Furthermore, the high frequency region demonstrates notable variation of the 1598/1627 cm^{-1} intensity ratio values, mainly due to cinnamaldehyde that exhibits its strongest scattering activity at this range. Hence, Raman spectroscopy appears to be sensitive to the presence of cinnamaldehyde due to its high Raman scattering cross section as a conjugated molecule, even though its relative concentration in cinnamon is low.

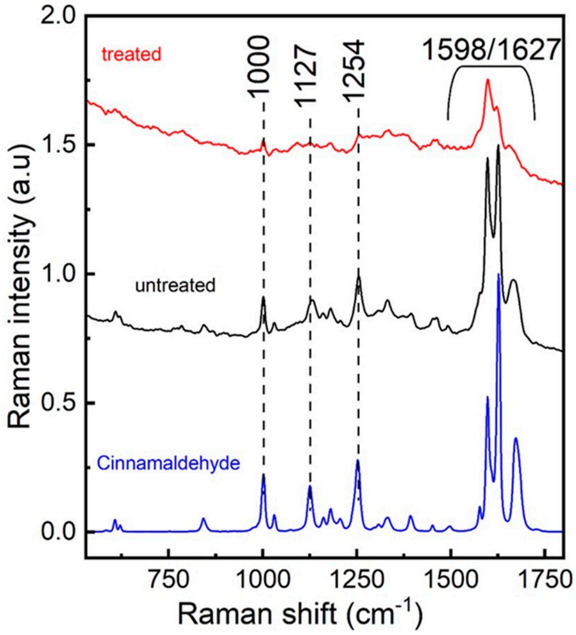

Figure S3. Raman spectra of pure cinnamaldehyde, untreated and treated cassia cinnamon.

Overall, the comparison of the two plots reveals that Raman spectroscopy is more sensitive than IR in probing the presence of cinnamaldehyde, which constitutes the main chemical marker found in both Ceylon and cassia cinnamon. Consequently, low or absent cinnamaldehyde content in either Ceylon or cassia labelled samples is strong evidence of suspicious cinnamon as already shown in Figs 4a and 5, which was attributed to the substitution by cinnamon camphora, other parts of the plant (e.g. root) and/or inorganic matter, as demonstrated by GC-MS and XRF analyses respectively. As a result, Raman spectroscopy is perceived as an efficient analytical tool for the detection of this type of cinnamon substitution that has never been documented in literature.
